# Engineered biomembrane-derived nanoparticles for nanoscale theranostics

**DOI:** 10.7150/thno.76894

**Published:** 2023-01-01

**Authors:** Ziqing Wu, Hao Zhang, Jing Yan, Yan Wei, Jiacan Su

**Affiliations:** 1 Institute of Translational Medicine, Shanghai University, Shanghai 200444, China.; 2 Institute of Medicine, Shanghai University, Shanghai 200444, China.; 3 Musculoskeletal Organoid Research Center, Shanghai University, Shanghai 200444, China.; 4 Department of Trauma Orthopedics, Changhai Hospital, Naval Medical University, Shanghai, 200433, China.

**Keywords:** biomembrane-derived nanoparticles, biomimetic, biomembrane engineering, targeted drug delivery, nanoscale theranostics

## Abstract

Currently, biological membrane-derived nanoparticles (NPs) have shown enormous potential as drug delivery vehicles due to their outstanding biomimetic properties. To make these NPs more adaptive to complex biological systems, some methods have been developed to modify biomembranes and endow them with more functions while preserving their inherent natures. In this review, we introduce five common approaches used for biomembrane decoration: membrane hybridization, the postinsertion method, chemical methods, metabolism engineering and gene engineering. These methods can functionalize a series of biomembranes derived from red blood cells, white blood cells, tumor cells, platelets, exosomes and so on. Biomembrane engineering could markedly facilitate the targeted drug delivery, treatment and diagnosis of cancer, inflammation, immunological diseases, bone diseases and Alzheimer's disease. It is anticipated that these membrane modification techniques will advance biomembrane-derived NPs into broader applications in the future.

## Introduction

Nanoparticles (NPs), made of nanoscale and biocompatible materials, can be utilized for targeted drug delivery and theranostic purposes 1. Generally, NPs can be divided into three categories: lipid-based NPs, polymeric NPs and inorganic NPs 2-6. According to different structures, each class of NPs can be further subdivided into different subclasses. For example, lipid-based NPs can be classified into liposomes 7-9, lipid NPs 10 and so on 2. NPs are able to encapsulate multiple drugs to increase their solubility and stability. Moreover, to achieve prolonged blood retention, the surfaces of NPs are always modified with hydrophilic polymers such as polyethylene glycol (PEG) 11, poly(2-oxazoline) (POx) 12 and poly(carboxybetaine) (PCB) 13. PEG is the most widely used material 14. Recognized as foreign objects, NPs are readily eliminated from blood circulation by cells from the mononuclear phagocyte system (MPS). The modification of NPs with PEG can generate a hydrated layer around the NPs to sterically shield their surface from aggregation, opsonization and phagocytosis, thereby prolonging the circulation half-life 15-18. However, the PEGylated NPs bind immunoglobulins on reactive B cells in the splenic marginal zone and thus stimulate the production of anti-PEG IgM. Upon the administration of a second dose, the previously generated anti-PEG IgM binds to PEG on the NPs and subsequently activates the complement system, leading to enhanced phagocytosis by Kupffer cells 19-21. This unexpected immunogenic response to PEG is commonly known as the “accelerated blood clearance (ABC) phenomenon”, which induces the increased clearance and reduced efficacy of PEGylated NPs.

To compensate for the deficiencies of artificially synthesized NPs, biomimetic NPs are becoming a research focus. In 2011, Zhang's team first synthesized red blood cell (RBC) membrane-camouflaged NPs 22. This reported top-down approach directly transferred RBC membranes onto biodegradable polymeric cores, by which the lipids, proteins and carbohydrates on the membrane could be retained to inherit the inherent biological properties of the source cells. Invaders are recognized by the immune system as foreign if they lack “markers of self” that are normally present on host cells or they express determinants that are absent. The RBC membrane surface protein CD47 (integrin-associated protein) functions as a “marker of self” and thus decreases the immune reaction to dramatically prolong the NP half-life to approximately 40 h 23. Compared with the 15.8-h half-life of PEGylated NPs, the RBC membrane camouflage provides an unusual advantage in extending the blood retention of the NPs. Thereafter, owing to the excellent outcomes, a variety of other biological membranes derived from cancer cells, white blood cells, platelets, and even exosomes have been used to prepare biomimetic NPs. Compared to artificially synthesized NPs, these biomimetic NPs have markedly improved drug delivery efficiency for superior efficacy in the treatment of cancer, inflammation, and immune diseases 24-27.

**Figure 1:**
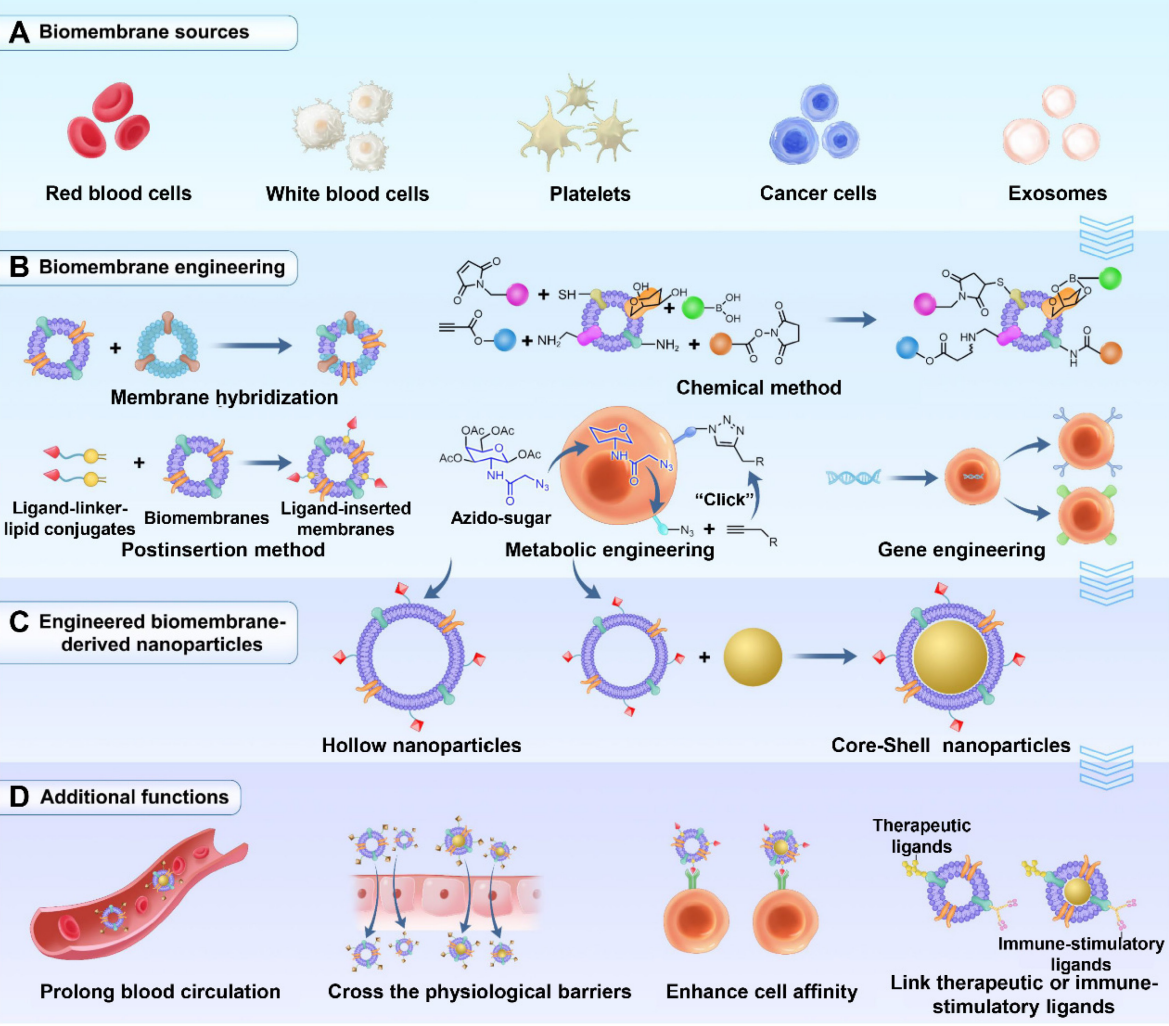
** Schematic illustrations of five membrane engineering methods and introduced additional functions. (A)** Different types of membrane materials used for engineering. **(B)** Membrane engineering methods: membrane hybridization, postinsertion method, chemical method, metabolic engineering and gene engineering. **(C)** Engineering biomembrane-derived nanoparticles: hollow or core-shell types. **(D)** Additional functions introduced by membrane engineering.

However, with the development of membrane-derived NPs, it was found that coating with a single membrane leads to certain limitations in the function of the NPs. For example, RBC membrane-coated NPs can reduce macrophage uptake, leading to a prolonged circulation time. However, their low targeting ability limits their therapeutic effects 28. Although macrophage membrane-coated NPs can escape phagocytosis by Kupffer cells to reduce liver uptake and aggregate at neuronal mitochondria, their cellular uptake is still insufficient 29. While certain types of exosomes can cross the blood-brain barrier (BBB), they lack target activity 30. Therefore, to meet the demands of drug delivery in complex situations, many research groups have begun to modify biological membranes to endow NPs with more functions. In this review, several frequently used modification methods of biological membranes, including membrane hybridization, the postinsertion method, chemical methods, metabolic engineering and gene engineering (Figure 1), are introduced. Furthermore, the review highlights examples of how the new functions were introduced through modification methods and worked with the inherent functions of source cell membranes to achieve better effects in the therapy and diagnosis of cancer, inflammation, immune diseases, bone diseases and Alzheimer's disease.

## Methods of fabricating biomembrane-derived NPs

The frequently used methods to fabricate biomembrane-derived NPs include the following three steps: membrane extraction, the preparation of NP cores and the coating of the NP cores with the membrane-derived vesicles (Figure 1C) 31. Unlike core-shell NPs, the procedures for the preparation of hollow NPs do not contain the last two steps. In addition, the methods of synthesizing NP cores often depend on the core materials and personalization, which are not reviewed here.

### Biomembrane extraction methods

The approaches to separating and extracting biomembranes depend on whether the cell has a nucleus. Nucleus-free cells, such as RBCs, can be lysed with hypotonic solution or repeated freeze‒thaw processes. Then, the membrane debris is isolated from the cell contents by centrifugation. Regarding nucleated cells, such as cancer cells and macrophage cells, the membrane isolation and extraction process is more complicated. In addition to hypotonic treatment, the cell structures need to be further destroyed *via* mechanical forces such as sonication and homogenization. Then, the cell membranes are isolated from the mixture containing nuclei and organelles *via* high-speed differential centrifugation 32-36.

In addition, exosomes are usually obtained as complete vesicles by ultracentrifuging the supernatants of the cell culture medium 37,38. Recently, microfluidic technologies have been developed for exosome separation 39-41. The passive microfluidic technique implements separation by imposing elastic lift forces on particles in viscoelastic media. The active microfluidic approach can separate exosomes according to their size and dielectric property by imposing acoustic radiation forces or spatially nonuniform electric fields. Compared to ultracentrifugation, microfluidic technologies collect exosomes with higher purity and a higher recovery rate.

### Biomembrane coating methods

Biomembrane-derived NPs can be fabricated by different methods, such as coextrusion, sonication, microfluidic electroporation or sonication, and *in situ* packaging methods. Physical extrusion relies on a mechanical force to destroy the membrane structure, thus enabling it to coat the core of NPs 32,42. The sonication approach exploits ultrasonic energy to assemble components into core-shell structures, which induces less material loss than coextrusion 33. In addition, microfluidic electroporation or sonication have emerged as novel approaches that utilize an electric pulse between two electrodes or sonication energy to promote the coating of nanocores with biomembrane vesicles. Biomembrane-derived NPs prepared by microfluidic techniques show better colloidal stability and higher coating efficiency than those fabricated with traditional extrusion 43.

In addition to utilizing purified membrane materials, there appears to be a unique technique that utilizes live cells to package core-shell NPs *in situ*. In this fabrication approach, when cells are incubated with gold NPs, iron-oxide NPs, or quantum dots, they can release vesicles containing these exogenous NPs 44.

## Methods of engineering biological membranes

### Physicochemical methods

#### Membrane hybridization

Membrane hybridization refers to hybridizing two different types of biological membranes or biological membranes and liposomes into a mixed membrane (Figure 1B). Since the biological functions of the membrane are derived from its source cells, the cell membranes should be carefully selected according to the characteristics of the origin cells and the demands for disease theranostics.

Two kinds of membranes can be easily hybridized by fusion 31,45. There is also an alternative process that hybridizes living cells and then extracts the fused membranes. Through this approach, the fused membranes can also simultaneously obtain the individual characteristics of two different cell membranes 46.

In addition to the hybridization of biomembranes and biomembranes, liposomes can also be fused with biomembranes. For example, the freeze-thaw method was used to fuse exosome membranes and liposomes, which can modulate the interplay between engineered exosomes and cells by altering the lipid components or the characteristics of exogenous lipids 47.

#### Postinsertion method

The postinsertion method is a nondestructive method for the physical insertion of functional ligands into biomembranes, which avoids damaging the membrane structure (Figure 1B). This modification technique utilizes the fluidity of cell membranes and the interaction between the hydrophobic interior of lipid bilayer membranes and the hydrophobic tails of ligands 48. The hydrophobic force controls the binding and direction of the inserted ligands and binds them to the membranes 49.

To insert the functional moieties, ligand-linker-hydrophobic anchor conjugates must first be synthesized. Hydrophobic tethered ligands are spontaneously embedded in the extracted biological membranes 50,51. Then, the ligand-decorated membrane materials can be coated on the nano cores (Figure 1B and 1C). During modification, sonication or extrusion is the frequently used method to facilitate ligand insertion. The postinsertion method is simple and robust and can control the ligand quantity by adjusting the lipid-tethered ligand input, providing versatility for platform optimization 52.

### Chemical methods

Chemical methods are convenient for modifying biological membranes with functional ligands (Figure 1B). With different covalent methods, moieties can be connected onto the membrane surface by coupling with functional groups on the membranes. For example, the natural amine groups on cell membranes can form an amide bond with the functional active ester groups of ligands 53. Other cell surface groups, such as cis-configured diols of polysaccharides, can also be exploited to form covalent linkages with boronic acid 54.

Chemical methods to engineer biological membranes have been developed recently. However, the application of chemical methods is limited because their harsh reaction conditions may destroy cell viability or membrane structure; therefore, more detailed characterization and method improvement are needed 55.

### Biological engineering

#### Metabolic engineering

Metabolic engineering is a flexible and versatile method that utilizes natural biosynthetic pathways to express ligands on biomembranes without destroying the membrane structure (Figure 1B). This approach mainly involves bioorthogonal chemistry 56,57. Briefly, the functional portions are first combined with artificial metabolic substrates such as saccharides. After incubation with the cells, the reaction groups can be modified on the membrane surface, as the given nonnatural substances can hijack the biosynthesis process.

After the membranes are extracted, bioorthogonal chemistry occurs in the next step to connect moieties. The reaction groups preintroduced onto the cell membranes can specifically and rapidly interact with the reaction groups on the functional ligands under mild physiological conditions. For example, azide groups can be modified onto cell membranes through glucose metabolism. By virtue of the biological orthogonal reaction of azide groups and bicyclo[6.1.0]nonyne (BCN), functional ligands containing BCN groups can be easily connected to cell membranes 58-60.

#### Gene engineering

Gene engineering uses biological tools to selectively modulate cell genes to modify the needed proteins or antibodies on the cell membranes (Figure 1B). Then, these engineered cell membranes can be extracted to wrap the nano cores.

Currently, there are many developed methods to carry genes into cells. Adenoviruses and retroviruses are the most common vehicles, but they possess undesirable disadvantages, such as viral toxicity and host immune rejection. Physical methods include gene gun bombardment 61, electroporation 62, ultrasonic energy 63, laser irradiation 64, and magnetic induction 65. Physical methods can stimulate the plasma membrane barriers to open relatively quickly, but it is difficult to quickly transfer DNA from the cytoplasmic region to the nucleus 66,67.

Clustered regularly interspaced short palindromic repeats (CRISPR)/CRISPR-associated protein 9 (CRISPR/Cas9) is becoming a dominant gene engineering tool in multiple organisms. Cas9 is an RNA-guided DNA endonuclease that is easily designed to target different sites by changing its guide RNA sequence. When the guide RNA recognizes a specific sequence in the genome, the Cas9 protein subsequently cuts the DNA sequence. During the process of DNA repair, an insertion or site-directed mutation can be introduced. Therefore, it is easy for CRISPR/Cas9 combined with gene delivery systems to delete, integrate and replace genes and thus enable the cells to express the needed proteins on their membrane surface 68.

## Engineered biological membrane-derived NPs

### Engineered red blood cell membrane-derived NPs

Red blood cells (RBCs) are the most abundant cells in the human body and are responsible for the transport of oxygen and CO_2_ 69. RBCs live up to 120 days in the body because their surface marker, CD47, can be linked to the inhibitory receptor signal regulatory protein alpha (SIRPα) and thus decrease immune elimination 70. Hence, the most prominent feature of RBCs is their prolonged circulation time. In addition, macrophages of the MPS are an effective part of destroying aged or abnormal RBCs. Therefore, NPs can be camouflaged with abnormal RBC membranes to target the MPS 71. As RBCs lack nuclei and most organelles, the RBC membrane extraction procedure is simpler than that of nucleated cells 72. Examples of RBC membrane engineering methods and introduced additional functions are summarized in Table 1.

Although RBC membrane-coated biomimetic NPs possess a long circulation ability, they lack sufficient targeting activity. Folic acid (FA) was first used to functionalize RBC membranes by postinserting FA-PEG-lipid conjugates into the membranes 52. The FA-modified RBC membrane-coated NPs could target tumor cells overexpressing FA receptors and significantly minimize off-target side effects 52,73. Stroke homing peptide (SHp) can effectively target the ischemic stroke site, which was selected to modify RBC membranes *via* the postinsertion of SHp-PEG-1,2-distearoyl-sn-glycero-3-phosphoethanolamine (SHp-PEG-DSPE). The resultant SHp-modified RBC membrane-coated NPs could markedly prolong the systemic circulation of the encapsulated neuroprotective agent NR2B9C and improve its active targeting toward the ischemic area in rats with middle cerebral artery occlusion, thereby decreasing ischemic brain lesions 74.

**Figure 2:**
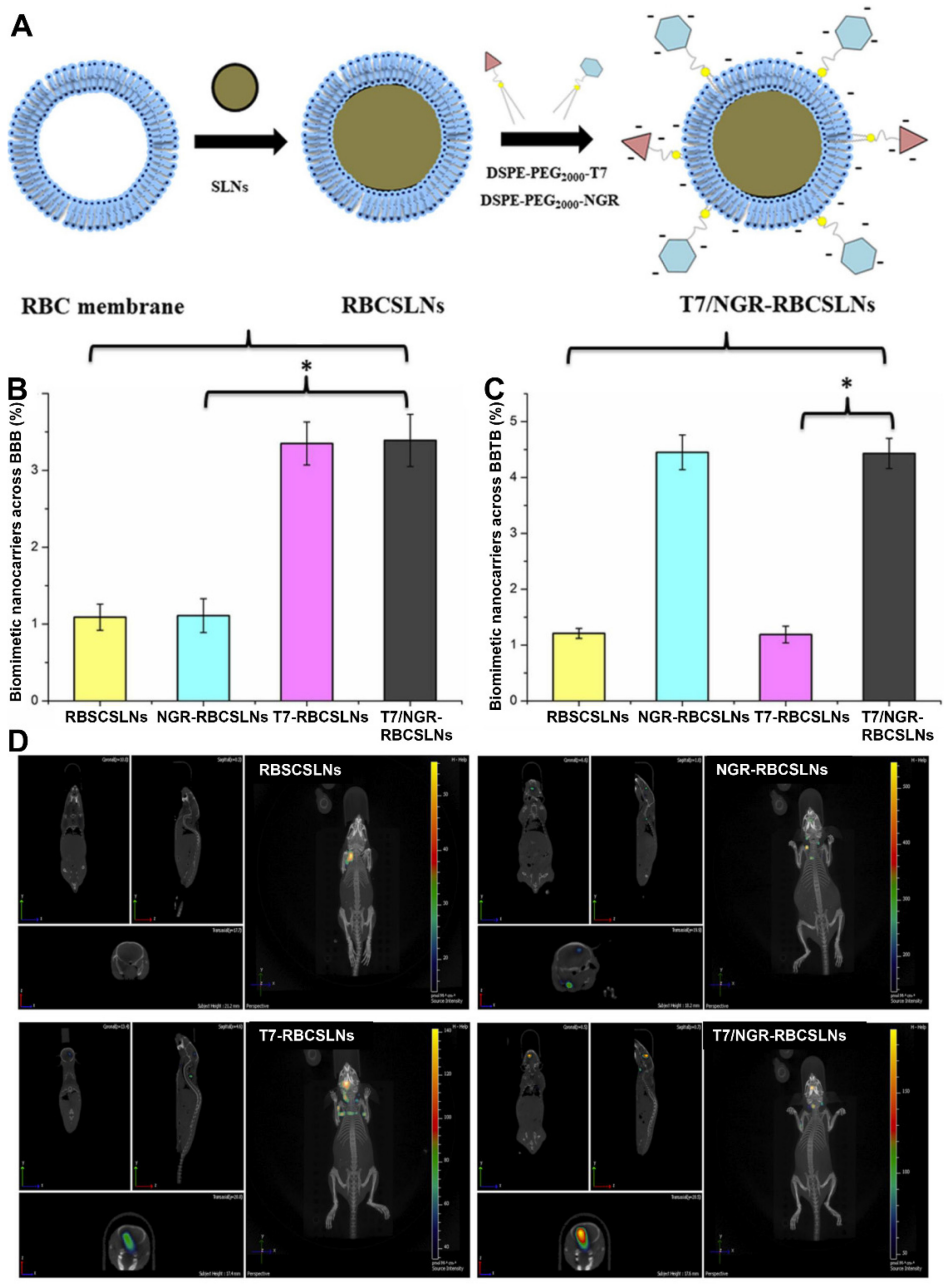
** Dual-modified RBC membrane-camouflaged solid lipid NPs (T7/NGR-RBCSLNs) for enhanced glioma targeting. (A)** Schematic illustration of the preparation procedure of T7/NGR-RBCSLNs. Penetrating efficiency of the indicated NPs in the *in vitro* models of the **(B)** blood-brain barrier (BBB) and **(C)** blood-brain tumor barrier (BBTB). **(D)** Biodistribution of the indicated Cy5.5-loaded NPs in glioma-bearing mice. Adapted with permission from 75, copyright 2018, American Chemical Society.

In addition to enhancing targeting ability, ligand modification can also promote RBC membrane-coated NPs to cross physiological barriers. It remains extremely challenging to deliver drugs across the BBB and blood‒brain tumor barrier (BBTB). The T7 peptide possesses a strong affinity for transferrin receptors (TfRs), which are highly expressed on the surface of the BBB. The NGR peptide can selectively bind to CD13, which is overexpressed during angiogenesis. As such, RBC membrane-camouflaged solid lipid NPs (RBCSLNs) decorated with T7 and NGR peptide (T7/NGR-RBCSLNs) were designed (Figure 2A). With the help of both T7 and the NGR peptide, T7/NGR-RBCSLNs could traverse the *in vitro* BBB and BBTB barriers (Figure 2B and 2C) and exhibited the highest accumulation in the brain tumor sites in glioma-bearing mice (Figure 2D). Finally, T7/NGR-RBCSLNs markedly improved the delivery and antiglioma efficacy of the loaded vinca alkaloid vincristine 75.

Similarly, RBC membrane-coated NPs modified with ^D^CDX (^D^CDX-RBCNPs) could also traverse the BBB and accumulate at the tumor site, as ^D^CDX exhibits strong affinity for nicotinic acetylcholine receptors expressed on the brain endothelium surface. The strong binding affinity of streptavidin to biotin was leveraged to construct ^D^CDX-RBCNPs. Briefly, after streptavidin was preimbedded into the RBC membrane surface, biotinylated^ D^CDX was efficiently incorporated into the NP surface. This modification approach avoids positively charged peptide interactions with negatively charged RBC membranes. With enhanced brain targeting efficiency, ^D^CDX-RBCNPs loaded with doxorubicin exerted excellent therapeutic efficacy in glioma-bearing mice 76.

### Engineered white blood cell membrane-derived NPs

White blood cells (WBCs) are immune cells that are responsible for protecting the body from infection, engulfing foreign bacteria and repairing tissue injury 77. Membranes from certain types of WBCs, such as T cells, macrophages, neutrophils, and dendritic cells (DCs), have widely been used to fabricate biomimetic NPs 78. Compared with bare NPs, WBC membrane-coated NPs can reduce opsonization and leverage the self-recognition mechanism of the WBC membrane to delay phagocytic uptake 79. In addition, WBC membrane-coated NPs inherit surface ligands from the WBCs that selectively bind to receptors at disease sites. Both of these features enable them to deliver drugs efficiently. Moreover, WBC membrane-camouflaged NPs can simulate WBC-cancer interactions to promote anticancer immunity 80. Examples of WBC membrane engineering methods and introduced additional functions are summarized in Table 1.

Owing to the specific immune recognition proteins expressed on the T-cell surface (e.g., T-cell receptors), activated T cells are able to recognize associated molecules on tumor cells, presenting inherent and strong tumor affinity. However, the natural tumor targeting of T cells is compromised by the intra- and interheterogeneity of tumors 81. As a complement, metabolism engineering can be utilized to make artificial receptors on the cell surface. After preconditioning T cells with Ac4GalNAz, the T-cell membranes expressed azide groups and were extracted to be coated on the indocyanine green NPs. The *in vivo* tumor cells were modified with BCN groups *via* intratumoral administration of AC_4_mann-BCN, whose metabolism would attach BCN to the membrane surface. Then, BCN can serve as an excellent targeting tag to chemically react with azide groups. Compared to unmodified T-cell membrane-coated NPs, the azide-modified T-cell membrane-coated NPs showed 1.5-fold higher tumor accumulation owing to specific binding to artificial BCN receptors on tumor cells 82. Ultimately, the azide-functionalized biomimetic NPs drastically increased photothermal therapy (PTT) efficacy without causing obvious side effects.

**Figure 3:**
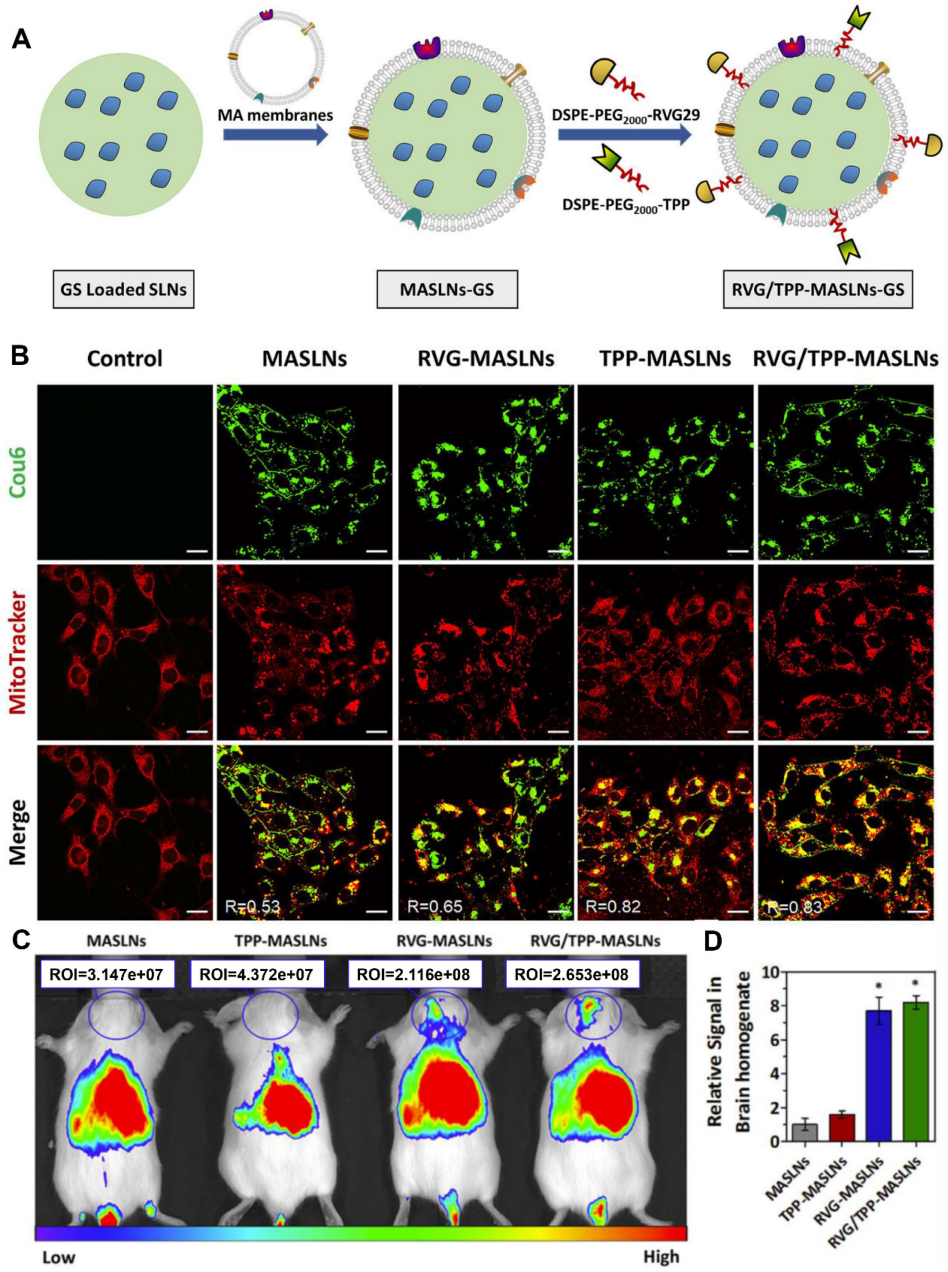
** RVG and TPP comodified macrophage membrane-coated solid lipid NPs loaded with genistein (GS) (RVG/TPP-MASLNs-GS) for enhanced neuronal mitochondria targeting. (A)** Preparation procedure of RVG/TPP-MASLN-GS. **(B)** Colocalization of different coumarin 6 (Cou6)-labeled NPs (green) with mitochondria (red) in differentiated HT22 cells.** (C)** Biodistribution of the various DiR-labeled NPs in the mice and** (D)** relative fluorescence signal of brain homogenate after bioimaging. Adapted with permission from 29, copyright 2020, Elsevier B.V.

Macrophages can be recruited to the inflammatory region. For example, inherent inflammation-oriented chemotaxis can direct macrophage membrane-coated NPs to accumulate in regions of the brain chronically inflamed owing to neurodegenerative diseases 83. On this basis, to improve the delivery efficiency for BBB crossing and neuronal targeting, the surface of the macrophage membrane was simultaneously modified with rabies virus glycoprotein (RVG29) peptide and positively charged triphenylphosphine (TPP) *via* the postinsertion method (Figure 3A) 29. RVG29 modification facilitated NP crossing of the BBB and internalization into neurons. The positively charged TPP ligand endowed the NPs with the mitochondrial-targeting property by leveraging negative mitochondrial membrane potential. As shown in Figure 3B, TPP modification promoted comodified NPs (RVG/TPP-MASLNs) to localize to the mitochondria of HT22 cells. Furthermore, with the ability to traverse the BBB barrier and accumulate in neurons, the dual-peptide-endowed NPs, RVG/TPP-MASLNs, displayed the most intense distribution in the brains of the mice (Figure 3C and 3D). Ultimately, genistein-loaded RVG/TPP-MASLNs were found to effectively delay the progression of Alzheimer's disease.

The neutrophil surface marker lymphocyte function-associated antigen 1 (LFA-1) can trigger the clustering of intercellular adhesion molecule 1 (ICAM-1) on the inflamed endothelium, locally improve vascular permeability, and extravasate across the inflamed endothelial layer. Because of their inherited source cell function, neutrophil membrane-coated NPs readily accumulate in the tumor microenvironment 84. However, they lack sufficient tumor cell internalization. Therefore, Wang's team modified paclitaxel-loaded neutrophil membrane-coated NPs with tumor necrosis factor-related apoptosis-inducing ligand (TRAIL) by chemical crosslinking that mediated internalization by binding with its receptors overexpressed on tumor cells 85. Finally, modification with TRAIL endowed the neutrophil membrane-coated NPs with 2-fold higher tumor accumulation and boosted their antitumor efficacy.

Mature antigen presenting cells (APCs) can handle certain antigens on tumor cell surfaces and activate cytotoxic T lymphocyte (CTL)-mediated anticancer immune responses. However, using natural APCs to expand and stimulate T cells* in vitro* is time-consuming and shows poor reproducibility 86. To resolve this dilemma, multifunctional artificial APCs (aAPCs) have been constructed 87. The DC membranes were premodified with azide by endogenous biosynthesis. Magnetic nanoclusters were wrapped with azide-decorated DC membranes and further linked with dibenzocyclooctyne (DBCO)-containing T-cell stimulus of peptide (SIINFEKL)-loaded major histocompatibility complex class-I (pMHC-I) and the costimulatory ligand anti-CD28 (αCD28) for EG-7 tumors by copper-free click chemistry. The resultant aPSCs induced effective amplification and stimulation of CTLs *in vitro* and visibly directed reinfused CTLs to the tumor site *via* magnetic resonance imaging and magnetic control. Ultimately, tumor growth in EG7 tumor-bearing mice was efficiently inhibited, indicating the great potential of this “one-but-all” aAPC platform for T-cell-based antitumor immunotherapy.

### Engineered cancer cell membrane-derived NPs

Since cancer cell membranes express both “self-markers” and “self-recognition” molecules, they can endow encapsulated NPs with prolonged systemic circulation and homotypic targeting capability 88,89. Additionally, cancer cells present various antigens on their surface, which could be leveraged to design biomimetic NPs for anticancer immune therapy 90. Examples of cancer cell membrane engineering methods and introduced additional functions are summarized in Table 1.

In addition to inherent homotypic targeting, the cancer cell membranes could be decorated with ligands to further augment their affinity for target cells. Inflamed endothelial cells highly express vascular cell adhesion molecule-1 (VCAM-1), which can recruit immune cells expressing its cognate ligand, very late antigen-4 (VLA-4). VLA-4 is a heterodimer that consists of integrin α4 and integrin β1. Wild-type C1498 leukemia cells highly express integrin β1 but lack integrin α4. Following viral transduction of C1498 cells with the integrin α4 gene, the resultant engineered cells expressed both VLA-4 components. The membranes from the genetically engineered cells were wrapped on the polymeric NP cores, and the resultant biomimetic NPs improved the delivery of the loaded anti-inflammatory drug dexamethasone to inflamed lung tissue and exerted important treatment efficacy *in vivo* 91.

**Figure 4:**
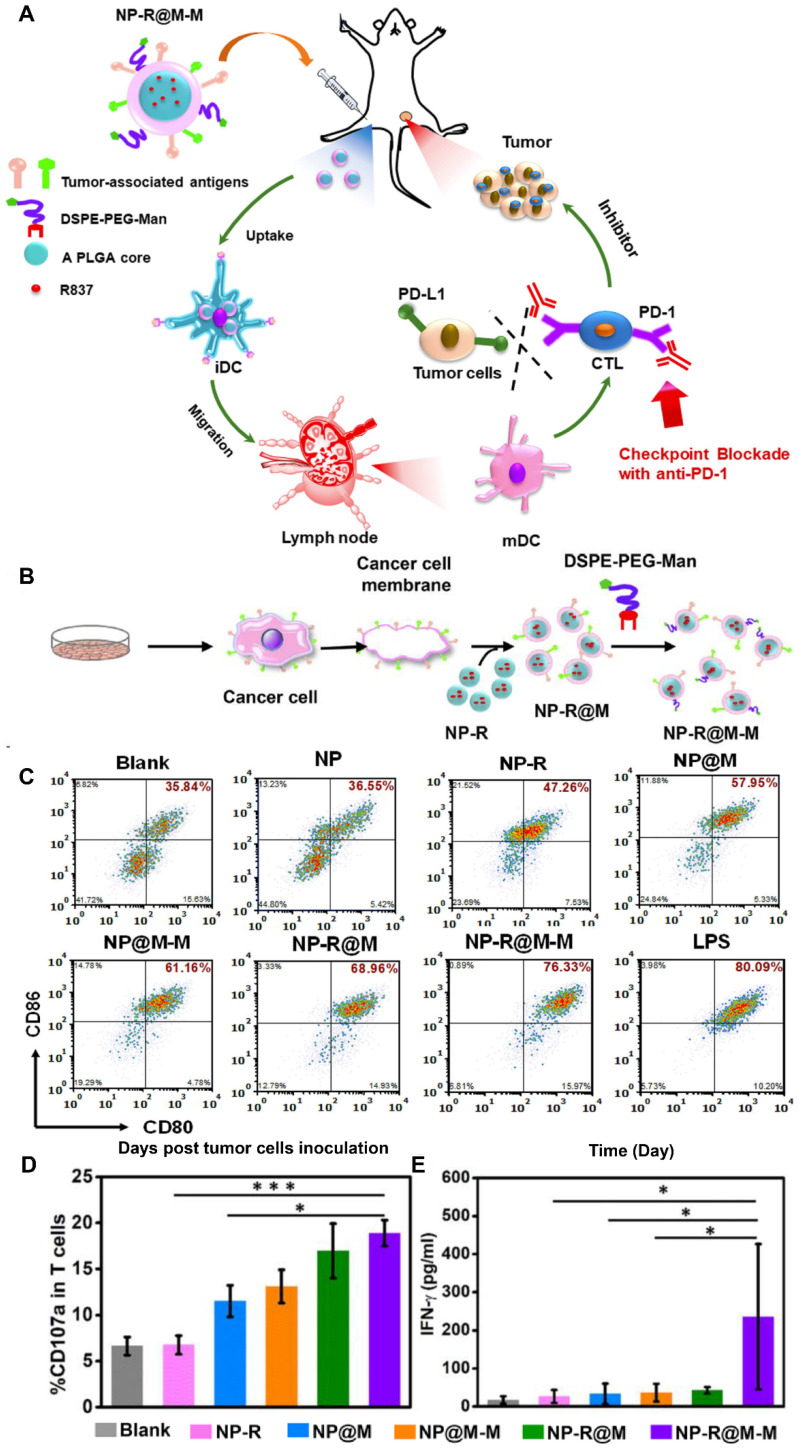
** Mannose-modified cancer cell membrane-cloaked PLGA NPs loaded with R837 (NP-R@M-M) acted as an effective anticancer vaccine. (A)** Proposed mechanism of action and **(B)** preparation procedure of NP-R@M-M. **(C)** Dendritic cell (DC) maturation after treatment with the indicated NP formulations. The cells were stained with CD11c antibodies as DC markers and CD80 and CD86 antibodies to label mature DCs. **(D)** The percentages of CD107a^+^ T cells determined by flow cytometry after intradermal injection with the different vaccine formulations. **(E)** IFN-γ concentration in sera drawn from the treated mice as determined by ELISA. Adapted with permission from 92, copyright 2018, American Chemical Society.

Cancer cell membranes expressing specific antigens on their surface can be exploited for the development of cancer vaccines. Decoration with additional ligands facilitates their affinity for immune cells. For example, NPs camouflaged with B16-OVA cancer cell membranes were further decorated with mannose through the postinsertion of DSPE-PEG-mannose (DSPE-PEG-Man) (NP-R@M-M), which can specifically bind to its receptors on DCs (Figure 4A and 4B) 92. Owing to enhanced internalization, treatment with NP-R@M-M induced highly effective DC maturation *in vitro* to a level comparable to that achieved by lipopolysaccharides (Figure 4C). In the immunized mice, NP-R@M-M induced the highest CD107a expression and interferon γ (IFN-γ) secretion (two typical markers of the cytotoxic activity of CTLs), effectively triggering an antitumor immune response (Figure 4D and 4E). Finally, NP-R@M-M alone acted as a prophylactic vaccine to inhibit tumor progression and effectively treated established B16-OVA melanoma tumors when it was combined with anti-programmed death-1 (anti-PD-1) checkpoint blockade.

In addition, cancer cell membranes can also be functionalized with additional ligands to facilitate their crossing of physiological barriers. For example, the cancer cell membrane could be modified with the cRGD peptide, which targets the integrin αvβ3 receptors highly expressed in the neovasculature, through metabolic engineering and a subsequent click reaction. Azide groups were introduced onto the glioma cell membrane by pretreating the cells with Ac_4_ManNAz. Then, the endo-BCN cRGD peptide was linked to azide groups on the membrane surface through the click reaction. The cRGD-decorated cell membrane was coated onto the nanocomposite core, comprising conjugated polymer (CP) and ultrasmall iron oxide (IO) nanoparticles (cRGD-CM-CPIO). With cRGD-mediated BBB crossing capabilities, cRGD-CM-CPIO exhibited significantly higher tumor accumulation than unmodified membrane-coated NPs (CM-CPIO) and was validated as an efficient imaging contrast agent 34.

In addition to improving the targeting ability, cancer cell membranes could also be decorated with a specific antibody to inhibit niche-mediated chemoresistance. Acute lymphoblastic leukemia (ALL) cells secrete pro-growth differentiation factor-15 (pro-GDF15) during chemotherapy, which can be cleaved by therapy-induced niche (TI-niche) cell-provided furin and subsequently activate TGF-β signaling to promote chemoresistance. The mesoporous silica NPs encapsulating daunorubicin (D@MSN) were cloaked with NALM-6 ALL cell membrane vesicles, which were preloaded with TGFβRII neutralizing antibody (aTGFβRII) through the postinsertion of its lipid conjugates incorporating a hypoxia-responsive azobenzene linker (DA_azo_@CMSN). DA_azo_@CMSN could home to the bone marrow TI-niche through the interaction of C-X-C motif chemokine receptor 4 (CXCR4, a chemokine receptor expressed on NALM-6 ALL CM) and stromal cell-derived factor-1 (SDF-1, a chemokine specifically secreted by bone marrow endothelial and stromal cells). Subsequently, aTGFβRII is detached from the NP surface *via* the hypoxic bone marrow microenvironment-mediated cleavage of the azobenzene linker and then inhibited GDF15-stimulated TGF-β signaling to block chemoresistance. Then, D@MSN could be taken up by ALL cells through homotypic targeting to enhance the chemotherapeutic efficacy of daunorubicin 93.

### Engineered platelet membrane-derived NPs

Platelets (PLTs) show a circulation half-life of approximately 30 h because they also express the “self-marker” of CD47 94, which endows PLT membrane-camouflaged NPs with prolonged blood retention ability. PLTs express a series of specific surface receptors that dynamically bind to damaged vasculature, pathogenic bacteria, and cancer cells 95-97. For example, PLT surface glycoprotein Ib (GPIb) can adhere to the exposed collagen of injured blood vessels via von Willebrand factor (vWF) for tissue repair 98. Furthermore, PLTs can link directly to pathogenic bacteria via GPIb to cause GPIIb/IIIa-mediated PLT aggregation as a portion of the host response to eliminate the bacteria 99. In addition, activated PLTs overexpress the cell-adhesion molecule P-selectin, which can combine with P-selectin glycoprotein ligand-1 (PSGL-1) or CD44 on tumor cells, leading to PLT-tumor interplay. The PLT membrane-derived NPs can replicate the adhesion functions from the source PLTs for active targeted drug delivery. For example, they can target damaged vasculature for the treatment of atherosclerosis, myocardial ischemia and pulmonary embolism; target drug-fast bacteria for the treatment of infectious diseases; and target primary tumors or circulating tumor cells (CTCs) for tumor therapy and detection. Examples of PLT membrane engineering methods and introduced additional functions are summarized in Table 1.

As PLT membrane-camouflaged NPs can target CTCs through membrane surface adhesion molecules, additional ligands can be introduced onto the membrane to kill CTCs. Biocompatible silica NPs were cloaked with the activated PLT membranes, followed by the conjugation of TRAIL onto the membrane surface by leveraging the affinity between streptavidin and biotin. The resultant biomimetic NPs could adhere to the CTC-induced thrombosis in the vasculature to deliver high-concentration cancer-killing TRAIL, thereby producing targeted therapeutic effects 100.

**Figure 5:**
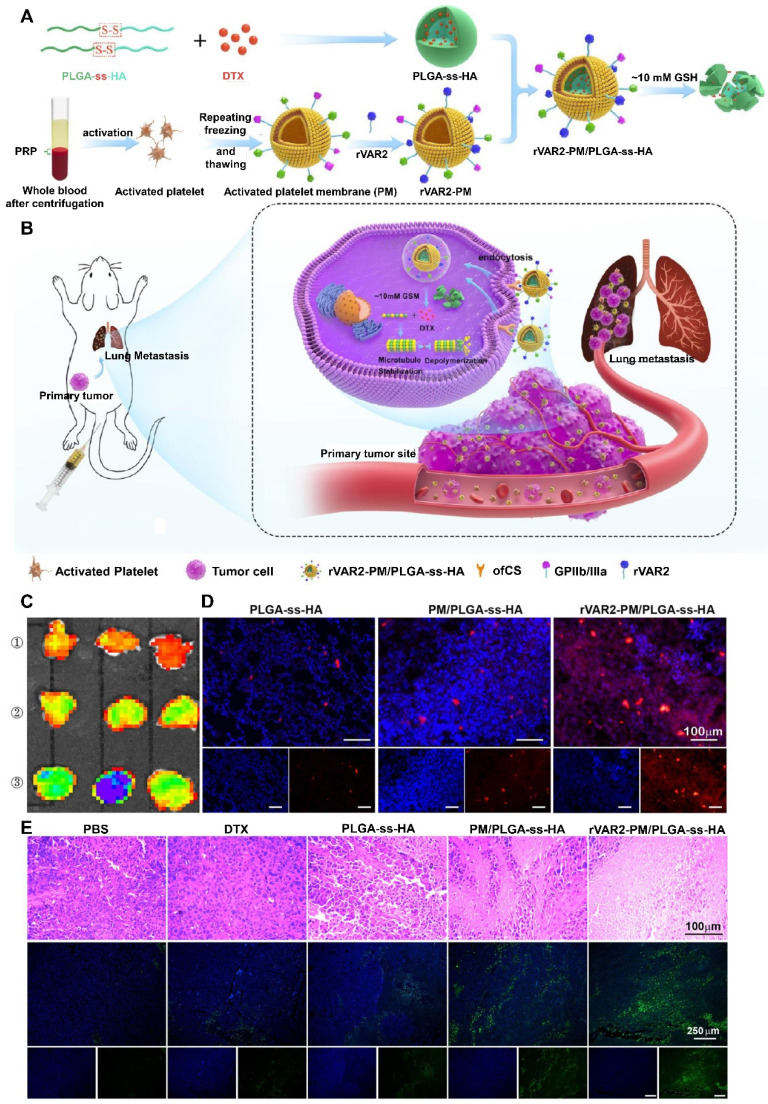
** The rVAR2 peptide-decorated activated platelet membrane-camouflaged PLGA-ss-HA NPs loaded with docetaxel (DTX) (rVAR2-PM/PLGA-ss-HA) for targeted therapy of primary and metastatic melanoma. (A)** Preparation procedure of rVAR2-PM/PLGA-ss-HA. **(B)** Proposed action mechanism of rVAR2-PM/PLGA-ss-HA. **(C)** *Ex vivo* fluorescence imaging of the excised B16-F10-bearing lungs at 4 h after injection with the indicated NPs. ①: PLGA-ss-HA; ②: PM/PLGA-ss-HA; ③: rVAR2-PM/PLGA-ss-HA. **(D)** Immunofluorescence staining of the excised B16-F10-bearing lungs. Red: DiD-labeled NPs; Blue: DAPI-stained cell nuclei. Scale bar: 100 µm. **(E)** H&E staining and TUNEL assay of the xenografts posttreatment with the indicated formulations. Adapted with permission from 101, copyright 2021, American Chemical Society.

Activated PLT membranes can also be engineered with additional ligands to strengthen their targeting efficiency to primary and metastatic tumors. For example, the recombinant VAR2CSA (rVAR2) peptide exhibited strong binding to oncofetal chondroitin sulfate (ofCS), which is selectively expressed on tumor cells. The rVAR2 peptide was introduced onto the activated PLT membranes through the postinsertion of DSPE-PEG-rVAR2, which were then coated on disulfide-containing PLGA-ss-hyaluronan (HA) NPs loaded with docetaxel (rVAR2-PM/PLGA-ss-HA) (Figure 5A and 5B) 101. Among all the treatment groups, rVAR2-PM/PLGA-ss-HA displayed the strongest accumulation into the lung metastasis foci of the mice, owing to both the activated PLT membrane and rVAR2-mediated active targeting (Figure 5C and 5D). Furthermore, H&E staining and TUNEL assays revealed that rVAR2-PM/PLGA-ss-HA led to the highest level of cell apoptosis, indicating effective inhibition of lung metastasis (Figure 5E).

### Engineered exosome membrane-derived NPs

Exosomes are extracellular vesicles released by all cells and range in size from 40 to 100 nm 102-104. Exosomes originate from the inward budding of the endosomal membrane, which will then invaginate to form intraluminal vesicles and further develop into multivesicular bodies (MVBs). Afterward, the MVBs probably fuse with the plasma membrane and release their vesicular content into the extracellular space as exosomes 105-107. During exosome formation, biomolecules such as cell-targeting ligands, cell adhesion molecules, and coding and noncoding RNAs can be loaded within the lipid bilayer or lumen 108. As such, the contents of an exosome are inherited from the source cells. Therefore, by carefully choosing the exosome source, exosomes can be utilized as a targeted drug delivery system. For example, brain endothelial cell-derived exosomes could cross the BBB in zebrafish embryos 109, and vascular endothelial cell-derived exosomes showed strong bone-targeting activity 110.

However, there still exist many limitations for exosomes as drug delivery tools. For example, despite possessing specific lipid and protein components, exosomes undergo rapid elimination from the blood circulation after intravenous injection 111. In addition, exosomes are nonspecifically distributed into unintended tissues such as the liver, spleen, and lungs 108. Fortunately, membrane engineering techniques provide an effective approach to compensating for the limitations of exosomes. Examples of exosome membrane engineering methods and introduced additional functions are summarized in Table 1.

Certain types of exosomes with a strong capability of crossing the BBB have been extensively studied for brain-targeted drug delivery but lack reliable targeting ability to brain tumors. Neuropilin-1 (NRP-1) is selectively expressed in glioma cells and the tumor endothelium and serves as an ideal target of glioma. To construct brain-targeted exosomes, superparamagnetic iron oxide NPs (SPION) and curcumin were encapsulated in the exosomes *via* electroporation. The exosome membranes were further modified with an alkyne group for subsequent binding of NRP-1-targeted peptide (RGERPPR, RGE) with azido groups. The resulting exosomes engineered *via* a chemical method could effectively traverse the BBB and accumulate in the glioma for enhanced imaging and treatment efficacy. Moreover, SPION-induced magnetic flow hyperthermia and curcumin-induced tumor growth inhibition also exerted strong synergistic antitumor efficacy 112.

**Figure 6:**
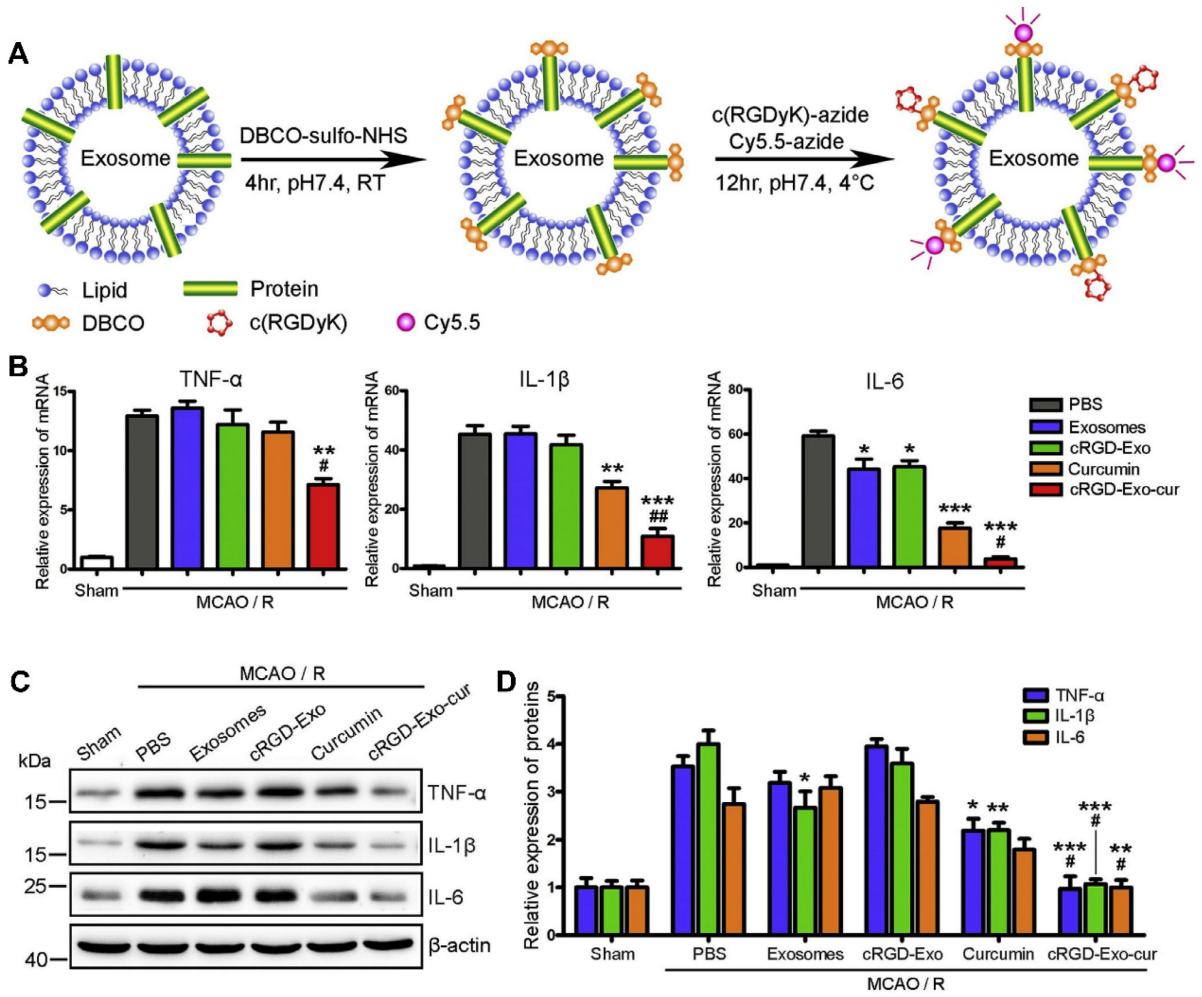
** c(RGDyK) peptide-modified exosomes as targeted drug delivery carriers for cerebral ischemia therapy. (A)** Procedure for the preparation of engineered exosomes with c(RGDyK) peptide. **(B)** Fluorescence imaging of the brain tissues of a transient middle cerebral artery occlusion mouse model at 6 h postinjection with the indicated formulations. **(C)** Quantitative assay of fluorescence intensity in the lesion region and **(D)** normalized ratios of fluorescence intensity in the ipsilateral versus contralateral region from Panel B. **(E)** Quantitation of the fluorescence intensity of various organs. Adapted with permission from 113, copyright 2017, Elsevier Ltd.

Similarly, DBCO groups could be incorporated into amine-containing molecules on exosomes *via* a heterobifunctional linker, which readily linked with azide-containing c(RGDyK) peptide *via* copper-free click chemistry (Figure 6A) 113. Then, curcumin was loaded into the engineered exosomes *via* incubation at room temperature (cRGD-Exo-cur). By exploiting the high affinity of the c(RGDyK) peptide for integrin αvβ3 on the reactive cerebral endothelium, the engineered exosome cRGD-Exo-cur displayed significantly higher accumulation in the lesion region than unmodified exosomes, especially with the fluorescence ratio of the ipsilateral to the contralateral region of 11 (Figure 6B-6E). cRGD-Exo-cur efficiently inhibited the inflammatory response and cellular apoptosis in a transient middle cerebral artery occlusion mouse model.

The targeting capability of exosomes can also be enhanced *via* gene engineering 114. Human embryonic kidney cell line 293 (HEK293) cells were transfected with pDisplay encoding GE11 to stably express the GE11 peptide. Hence, the exosomes secreted from the transfected cells highly expressed the GE11 peptide on their surface. By leveraging the specific binding activity of GE11 to EGFR, the engineered exosomes could transfer let-7a miRNA to EGFR-expressing breast cancer xenografts in RAG2 (-/-) mice after intravenous injection, effectively inhibiting tumor development *in vivo* 115. Based on the same principle, CXCR4 could be introduced onto exosome membranes by genetically engineering NIH-3T3 cells. Then, the CXCR4^+^ exosomes were fused with liposomes carrying antagomir-188 to prepare the hybrid exosomes. As SDF-1 is highly abundant in the bone marrow, the hybrid CXCR4^+^ exosomes selectively gathered in the bone marrow to release antagomir-188, promoting the osteogenesis of marrow mesenchymal stem cells and reversing age-related trabecular bone loss 38.

In addition to enhancing targeting ability, membrane engineering can also prolong the blood circulation of exosomes. Epidermal growth factor receptor (EGFR)-specific nanobodies could be combined with phospholipid (DMPE)-PEG derivatives to prepare nanobody-PEG-micelles. After incubation with exosomes from Neuro2A cells or PLTs, nanobody-PEG-micelles transferred to exosome membranes in a temperature-dependent manner. After the introduction of EGFR-specific nanobodies, the exosomes showed significantly increased binding ability to EGFR-overexpressing tumor cells. Furthermore, compared with the unmodified exosomes that were eliminated from the blood circulation of the mice within 10 min, exosomes decorated with nanobody-PEG-micelles were still detectable in plasma after 1 h. Therefore, the insertion of ligand-conjugated PEGylated phospholipids could endow exosomes with prolonged blood retention and enhanced cell specificity, effectively promoting exosome enrichment in targeted tissues for improved cargo delivery 116.

### Hybrid cell membrane-derived NPs

Monotypic cell membranes hardly meet the complex needs of drug delivery for specific diseases. In contrast, the hybridization of various kinds of cell membranes that inherit the specific properties from the source cells can endow the NPs with a variety of biofunctions, thereby demonstrating superior efficacy and safety 117-119. Examples of hybridized biomembrane-derived NPs are listed in Table 2.

### Hybridization with RBC membranes

As mentioned above, the RBC membranes from natural long-circulating vesicles can prolong the blood circulation time of the modified NPs. On this basis, another type of cell membrane can be hybridized with the RBC membrane to add new functions, e.g., homotypic targeting ability. For example, for combination treatment of melanoma, the membranes from RBCs and melanoma cells (B16-F10) were hybridized and wrapped on doxorubicin-loaded hollow copper sulfide NPs (DCuS@[RBC-B16]). RBC membranes markedly prolonged the blood retention of DCuS@[RBC-B16], with 20.2% ID/g remaining at 24 h postinjection, relative to 14.5% ID/g of DCuS@[B16] remaining. Furthermore, DCuS@[RBC-B16] NPs that retained the characteristics of B16-F10 cells exhibited strong specific self-recognition to the B16-F10 cells. Ultimately, owing to enhanced tumor-targeted delivery, DCuS@[RBC-B16] NPs exerted striking synergistic photothermal/chemotherapeutic effects, with an almost 100% growth inhibition rate of melanoma tumors 120.

Likewise, membranes originating from RBCs and the MCF-7 cancer cell line were hybridized and coated on melanin NPs (Melanin@RBC-M) for PTT of tumors. Interestingly, a higher MCF-7 membrane ratio led to a stronger homotypic targeting ability of Melanin@RBC-M, while a higher RBC membrane ratio induced longer blood retention. In MCF-7 tumor-burdened nude mice, Melanin@RBC-M with a 1:1 membrane protein weight ratio of RBCs to MCF-7 showed the highest tumor delivery and superior PTT efficacy when compared with other Melanin@RBC-M NPs with different ratios of RBCs to MCF-7 or pristine melanin NPs. This occurred because of the optimal balance between prolonged blood retention and homotypic targeting 28.

Based on the same principle, RBC membranes were mixed with retinal endotheliocyte (REC) membranes for coating onto PLGA NPs ([RBC-REC]NPs) 121. The RBC membranes enabled [RBC-REC]NPs prolonged blood retention, while the self-recognition capability of REC membranes enabled [RBC-REC]NPs to target RECs. In a laser-induced wet age-related macular degeneration mouse model, [RBC-REC]NPs effectively accumulated in the choroidal neovascularization area. Through the receptors VEGFR1 and VEGFR2 expressed on REC membranes, [RBC-REC]NPs could bind to VEGF-A ligands to block their effects on host endothelial cells, greatly reducing the choroidal neovascularization area and leakage.

PLTs can bind to exposed collagen in injured blood vessels, which enables the PLT membrane to actively target damaged vasculature 98. To this end, PLT membranes were hybridized with RBC membranes to wrap on polypyrrole nanoparticles (PPy@[R-P] NPs) 122. The resultant PPy@[R-P] NPs inherited the properties of RBCs and PLTs, indicating prolonged blood retention and self-targeting ability toward the injured vasculature. After intravenous injection with PPy@[R-P] NPs, tumor vessels were damaged through near-infrared laser exposure-induced photothermal stimulation, leading to extensive microthrombosis. The PLT membrane coating enabled numerous PPy@[R-P] NPs to be recruited to the microthrombosis sites, leading to impressive antitumor PTT efficacy.

### Hybridization with WBC membranes

In addition to RBC membranes, leukocyte membrane camouflage can also be utilized to escape immune clearance for prolonged blood retention and can be mixed with tumor cell membranes for additional homotypic targeting. For example, the membranes from macrophages (murine J774A.1 cells) and tumor cells (head and neck tumor HN12 cells) were fused with exogenous phospholipids, yielding leutusome 123. Herein, exogenous phospholipids were added as building blocks to aid the fusion of two cell membranes and the encapsulation of poorly water-soluble paclitaxel. With the combined aid of immune-evading and homotypic targeting ability of these two membranes, the leutusome obtained a prolonged plasma half-life of 8.1 h and markedly increased tumor accumulation. Finally, the leutusome exerted the most potent inhibition of tumor growth without causing systemic adverse effects.

**Figure 7:**
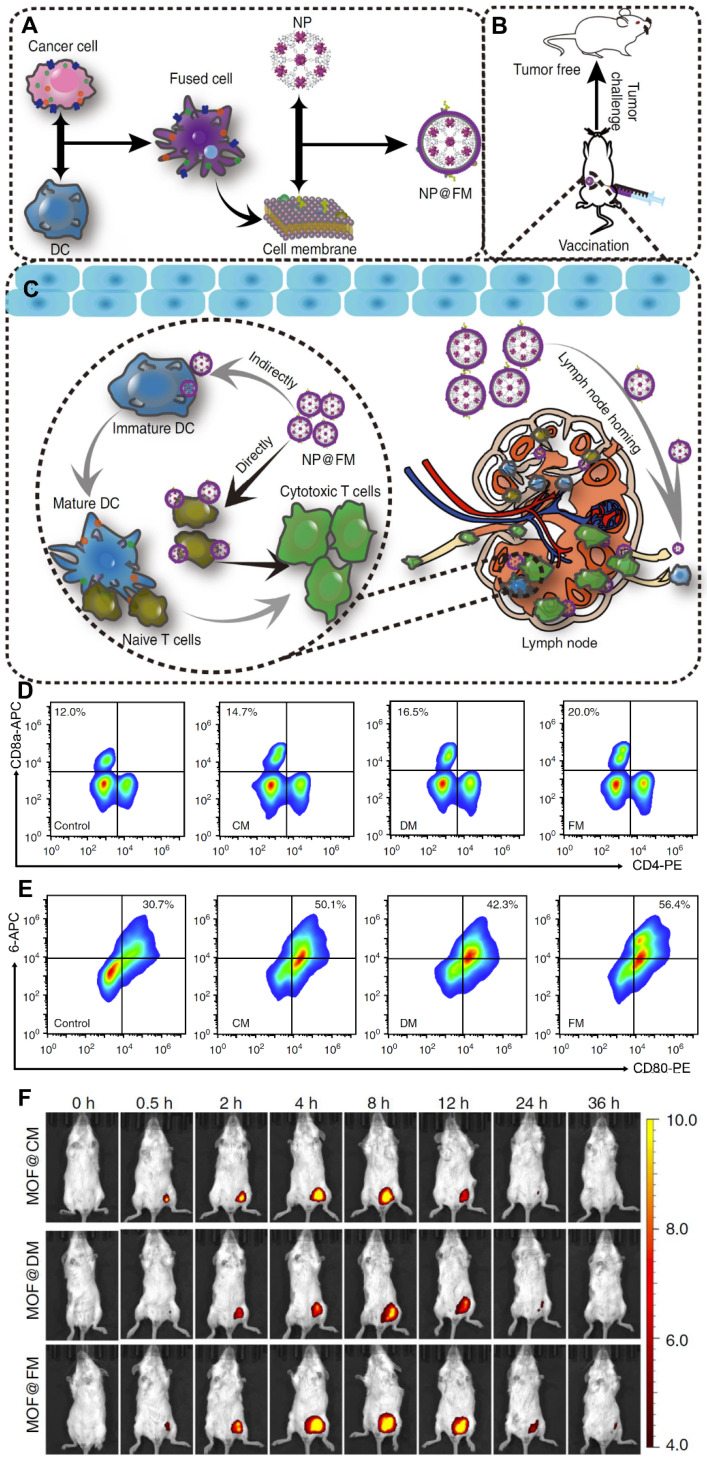
** Cell membranes from fused cells of cancer cells and dendritic cells (DCs) were coated onto metalorganic framework (MOF) NPs (NP@FM) for antitumor immune therapy. (A)** Preparation procedure of NP@FM. **(B)** Vaccination of NP@FM for cancer prevention.** (C)** Action mechanisms of NP@FM by directly and indirectly activating T cells to induce immune responses. **(D)** The expression of CD8 and CD4 (markers for T-cell activation) measured *via* flow cytometry after the incubation of T cells with the indicated cell membrane vesicles for 48 h. **(E)** The expression of CD80 and CD86 (markers for DC maturation) after the incubation of DCs with the indicated cell membrane vesicles for 48 h. **(F)** *In vivo* fluorescence imaging of mice after subcutaneous injection with the indicated NPs at a series of time points. Adapted with permission from 125, copyright 2019, Nature Communications.

In addition to drug delivery, membrane hybridization can also be leveraged to strengthen the antitumor immune response. DCs enable the uptake, processing and presentation of tumor antigens in the form of antigen peptides-major histocompatibility complexes (pMHCs) on their surface 124. Then, mature DCs prepare different subsets of antigen-specific T cells to attack and kill homologous tumor cells. Based on this principle, DCs and tumor cells were hybridized, and the fused cytomembranes (FMs) were extracted to be coated on the fluorescent metal-organic framework (MOF) NPs, yielding the nanovaccine (NP@FM) (Figure 7A) 125. Notably, the fusion of both of these immunologically related cells led to strong presentation of the whole tumor antigens and immunological costimulatory molecules on the membranes. On the one hand, the expression of immunological costimulatory molecules allowed NP@FM to directly activate T-cell immunity. On the other hand, tumor antigen-bearing NP@FM could be recognized by DCs, thereby resulting in DC-mediated indirect T-cell immunoactivation. After incubation *in vitro,* FMs induced stronger T-cell activation and DC maturation than cancer cell membranes or DC membranes (Figure 7B and 7C). As shown in Figure 7F, MOF@FM showed a better retention effect than MOF@DM or MOF@CM due to the effect of lymph node-tropic migration and location after DC maturation. This approach of combining direct T-cell activation and indirect DC-to-T-cell activation provided a robust antitumor immune response.

Certainly, all the mentioned membrane engineering technologies are not mutually exclusive and thus can be used together to obtain multiple advantages. For example, membranes derived from PLTs and WBCs were hybridized to be wrapped onto magnetic beads, and then their surface was decorated with antiepithelial cell adhesion molecules (anti-EpCAMs) that could recognize EpCAM-positive CTCs 126. Specifically, anti-EpCAM modification was achieved through step-by-step surface conjugation of the biomimetic NPs with DSPE-PEG-COOH, streptavidin, and biotinylated anti-EpCAMs. The resultant PLT-WBC hybrid membrane-camouflaged immunomagnetic beads (HM-IMBs) decreased homotypic WBC interactions from WBCs and inherited improved tumor cell binding capability from PLTs and anti-EpCAMs. Compared with commercial IMBs, the CTC separation efficiency and purity of HM-IMBs from spiked blood samples were improved from 66.68% to 91.77% and from 66.53% to 96.98%, respectively. Moreover, highly pure CTCs were successfully isolated using HM-IMBs from 19 out of 20 clinical blood samples from breast cancer patients. Hence, HM-IMBs represent a highly specific and efficient isolation approach for CTCs, thereby overcoming the restrictions of current theranostic platforms.

## Challenges

Although membrane engineering technology has shown promising outcomes in various fields, this technology is still in its initial stage and confined to laboratory research. Numerous challenges prevent its industrial process scale-up or clinical translation.

Each modification method has its inherent advantages and limits (Table 3). For example, ligands introduced onto membranes *via* the postinsertion method maintain the activity of membrane proteins, but the binding is less firm than when ligands are introduced through covalent reactions 127. However, the chemical method introducing ligands through covalent reactions may destroy the original active structure of the membrane due to its relatively harsh conditions 55. Moreover, although gene engineering introduces functional proteins with the right conformation and at specific sites, the procedure is cumbersome, and it is difficult to ensure stable expression of the target gene 128-130. In contrast, the engineering of membrane vesicles guarantees that a higher proportion of decorated molecules are located at the outer leaflets of NPs 131. Metabolic engineering introduces unnatural functional groups onto the membrane surface, but the groups have to be small and inert 132-134. Many factors, such as drug delivery needs and therapeutic purposes, should be considered to select a suitable modification method.

The cell membranes are a part of living entities. Therefore, during the modification process, the reaction conditions, such as the reaction temperature, the special reagents used, or the substrate concentration, may damage the activity of the cell membranes. However, there is a lack of necessary criteria or a suitable judgment basis to choose the reaction conditions. Furthermore, the characterization methods to verify successful membrane modification provide only very limited information. For example, particle size measurement and morphology observation make it difficult to recognize modified small molecules. Western blot analysis provides information only on the distinction of protein components between the engineered and the source cell membrane. However, it cannot evaluate whether the cell membrane activity is retained after subjection to the engineering process. Therefore, there is a great need to develop more sophisticated methods to improve the visualization of membrane engineering processes.

In addition, other existing challenges also prevent industrial process scale-up or clinical application. First, the extraction technologies should be optimized to acquire enough biomembranes, and tailored procedures are required to enhance the membrane purity from the nucleated cells. Second, strict quality control is required in every step involved in the preparation process of engineered biomembrane-derived NPs. For example, some conventional formulation parameters, such as drug encapsulation efficiency, drug content and drug release behavior, should be monitored. Furthermore, for the hybrid membrane-coated NPs, many points are worth considering, such as each membrane ratio, fusion efficiency and fused membrane stability. For NPs prepared from genetically engineered membranes, the expression level and practical effects of the exogenous components are significant in determining the final performance of NPs in disease therapy. As such, the identification of the moiety-presenting profile and level and evaluation system should be established in detail. Furthermore, the long-term storage stability of the biomimetic NPs should be fully studied and is worth further improvement.

## Conclusion and future perspectives

Modified biological membrane-derived NPs are becoming a research hotspot to resolve the limitations of single membrane-derived NPs. A variety of membrane modification methods, including membrane hybridization, the postinsertion method, chemical methods, metabolic engineering and gene engineering, endow biomimetic NPs with additional functions to meet the multiple requirements of drug delivery and disease therapy and diagnosis. Currently, numerous modified biological membrane-derived NPs have been developed and have shown encouraging outcomes in preclinical research.

However, membrane engineering technologies are still in their infancy. To realize scale-up production and clinical translation, there is still a long way to go. For example, necessary criteria to select the reaction conditions and sophisticated characterization methods to improve the visualization of the membrane engineering process should be established. Recently, Amitava Moulick *et al.* synthesized gadolinium-Schiff base-doped quantum dot (GdQD)-based probes for the fast, facile, spatial detection of membrane injuries 135. These probes function by preferentially interacting with NHE-RF2 scaffold proteins exposed after membrane damage. In addition, the membrane extraction technique, quality control of the preparation process and long-term storage stability of the NPs need to be further studied or optimized. For example, protein affinity purification-mass spectrometry and cell sorting technology are suggested for membrane purification methods that can analyze the unique antigens in membrane materials. Freeze-drying represents an effective method owing to the enhanced stability of solid formulations. Monica Guarro *et al*. used sucrose at 8.5 wt% as a lyoprotectant and improved the cryopreservation efficiency of exosomes, enabling the preservation of their physicochemical properties and functionality for a long time 136.

Despite much room for improvement, it cannot be overlooked that membrane engineering techniques can effectively functionalize NPs, thus enhancing their therapeutic or diagnostic effects. Overall, we strongly believe that these emerging biomembrane engineering approaches will acquire a wider application in solving pressing medical problems in the foreseeable future.

## Figures and Tables

**Table 1:** Summary of engineering methods of biomembranes and introduced additional functions

Source cells	Inherent functions	Engineering methods	Introduced additional functions	Particle size (nm)	References
Red blood cells	Prolong blood circulation time; Abnormal RBCs can target the mononuclear macrophage system.	Postinsertion method	Enhance targeting ability	75-200	52,73-76
			Cross physiological barriers		
T cells	Avoid being cleared by the immune system; Target tumor sites.	Metabolic engineering	Enhance targeting ability	75	82
Macrophage cells	Avoid being cleared by the immune system; Target inflammatory or tumor sites.	Postinsertion method	Cross physiological barriers; Enhance targeting ability	123	29
Neutrophil cells	Avoid being cleared by the immune system; Extravasate across inflamed endothelial layer.	Chemical method	Enhance targeting ability	70	85
Dendritic cells	Avoid being cleared by the immune system; Present antigens to stimulate an immune response.	Metabolic engineering	Link immune-stimulatory ligands	240	87
Cancer cells	Prolong blood circulation time; Homotypic targeting capability; Present antigens to stimulate an immune response.	Gene engineering	Enhance targeting ability; Cross physiological barriers; Link therapeutic ligands	84-175	34, 91-93
		Postinsertion method			
		Metabolic engineering			
Platelets	Prolong blood circulation time; Target damaged vasculature; Target pathogenic bacteria and cancer cells.	Chemical method; Postinsertion method	Link therapeutic ligands; Enhance targeting ability	106-122	100-101

Exosomes	The contents are inherited from the source cells; Certain types of exosomes can cross the blood-brain barrier.	Chemical method	Enhance targeting ability; Prolong blood circulation	113-143	38, 112-113, 115-116
		Gene engineering			
		Postinsertion method			

**Table 2:** Summary of membrane hybridization methods and introduced functions

Original cell membranes	Introduced cell membranes	Introduced functions	References
Red blood cell membranes	Cancer cell membranes	Add homotypic targeting ability; Add active targeting ability.	28,120-122
	Retinal endotheliocyte membranes		
	Platelet membranes		
White blood cell membranes	Cancer cell membranes; Platelet membranes	Add homotypic targeting ability; Add active targeting ability.	123, 126

**Table 3:** Advantages and disadvantages of different types of biomembrane engineering methods

Biomembrane engineering methods	Advantages	Disadvantages
Membrane hybridization	Combine the functions of different membranes	Types and proportions of membranes need to be explored
Postinsertion method	Without destroying cell viability or membrane structure	Binding of the ligands onto the membrane is less firm
Chemical methods	Easy to modify, and the binding of ligands onto membranes is solid	Possibly destroy cell viability or membrane structure
Metabolic engineering	Introduce unnatural chemical functional groups onto membrane surface	Introduce only small and biologically inert groups
Gene engineering	Introduce functional proteins with right conformation and at specific sites on membranes	The procedure is cumbersome, and it is difficult to ensure stable expression of the target gene
